# 

**Published:** 1948

**Authors:** 


### The Medical Library of the University of Bristol

WITH WHICH IS INCORPORATED

**The Library of the Bristol Medico-Chirurgical Society.**

---

*The following donations have been received since the publication of the list in the last issue.*

| | |
|---|---|
| General Medical Council (1) .. .. .. .. | 2 volumes |
| Dr. W. A. Gornall (2) .. .. .. .. .. | 13 volumes |
| Henry Phipps Institute (3) .. .. .. .. | 1 volume |
| J. Angell-James Esq. (4) .. .. .. .. | 1 volume |
| Michell Clarke Fund (5) .. .. .. .. | 10 volumes |
| Royal College of Physicians of London (6) .. .. | 1 volume |
| Philip Thursfield, Esq. (7) .. .. .. .. | 2 volumes |
| Transvaal Chamber of Mines (8) .. .. .. | 1 volume |
| U.N.E.S.C.O. (9) .. .. .. .. .. | 1 volume |
| University of Otago Medical School (10) .. .. | 1 volume |

Unbound periodicals have also been received from the British Medical Association, Dr. C. D. Evans, Professor T. F. Hewer, Professor A. V. Neale, Dr. J. C. G. Whitelaw and Messrs. John Wright & Sons Ltd.

### THE ONE HUNDRED AND EIGHTY-EIGHTH LIST OF BOOKS

The figures in round brackets refer to the figures after the names of the donors. The books to which no such figures have been attached have either been bought from the Library Fund or received through the Journal.

| | | |
|---|---|---|
| **Allen, E. V. and others** | *Peripheral Vascular Diseases* .. .. .. .. .. | 1947 |
| **Allison, D. R. and Gordon, R. G.** | *Psychotherapy—its Uses and Limitations* | 1948 |
| **Andrews, G. C.** .. .. | *Diseases of the Skin* .. .. .. 3rd Ed. (5) | 1947 |
| **Babington, C. C.** .. .. | *History of the Infirmary and Chapel of the Hospital and College of St. John the Evangelist at Cambridge* | 1874 |
| **Bailey, H.** .. .. .. | *Demonstrations of Physical Signs in Clinical Surgery Part* 1 .. .. .. .. .. .. 11th Ed. | 1948 |
| **Bailey, H.** .. .. .. | *Emergency Surgery Part* 1 .. .. .. 6th Ed. | 1948 |
| **Bainbridge, F. A. and Menzies, J. A.** | *Essentials of Physiology*, 9th Ed. by *H. Hartridge.* .. .. .. .. .. .. .. (5) | 1947 |
| **Barnes, T.** .. .. .. | *Observations on the Expediency and Advantage of Establishing a General Infirmary at Carlisle* 2nd Ed. | 1831 |
| **Begbie, J. W.** .. .. | *Sketch of the Early History of Anatomy* .. .. .. | 1868 |
| **Breen, G. E.** .. .. | *Essentials of Fevers* .. .. .. .. 2nd Ed. (5) | 1948 |
| **Brend, W. A.** .. .. | *Handbook of Medical Jurisprudence and Toxicology* .. .. .. .. .. .. .. 8th Ed (5) | 1941. |

| | | | |
|---|---|---|---|
| | *British Encyclopaedia of Medical Practice*: (1) *Medical Progress* 1948: (2) *Cumulative Supplement* 1948 | | 1948 |
| **British Medical Association.** | *The Training of a Doctor* | | 1948 |
| **Brockbank, E. M.** | *The Conduct of Life Assurance Examinations* | 2nd. Ed. | 1947 |
| **Brownlow J.** | *The History and Objects of the Foundling Hospital* | 3rd. Ed. | 1865 |
| **Castiglioni, A.** | *History of Medicine* | 2nd. Ed. (5) | 1947 |
| **Crowe, H. W.** | *Handbook of Vaccine Treatment of Chronic Rheumatic Diseases* | 3rd. Ed. (2) | 1939 |
| **East, T. and Bain, C.** | *Recent Advances in Cardiology* | 4th Ed. (5) | 1948 |
| **Edwards, H. C.** | *Recent Advances in Surgery* | 3rd Ed. | 1948 |
| **Francis, J.** | *Bovine Tuberculosis* | (5) | 1947 |
| **Franklin, K. J.** | *Cardiovascular Studies* | (5) | 1948 |
| **Greenwood, M.** | *Some British Pioneers of Social Medicine* | | 1948 |
| **Hall, I. S.** | *Diseases of the Nose, Throat and Ear* | 4th Ed. | 1948 |
| **Hamilton, W. J., and others** | *Human Embryology* | | 1946 |
| **Harvey, W.** | *Facsimile Reproduction of the Diploma of Doctor of Medicine granted by the University of Padua . . . with Notes by J. F. Payne* | (7) | 1908 |
| **Hawkins, R. R. (Ed.)** | *Scientific, Medical and Technical Books Published in U.S.A.* 1930-1944 | (9) | 1946 |
| **Hill, A. B.** | *Statistics in Medicine* | | 1947 |
| **Hutchison, R.** | *Food and the Principles of Dietetics: revised by V. H. Mottram and G. Graham* | 10th Ed. | 1948 |
| **Johnston, T. B.** | *Medical Applied Anatomy* | (2) | 1915 |
| **Kolff, W. J.** | *New Ways of Treating Uraemia* | | 1947 |
| **La Wall, C. H.** | *Four Thousand Years of Pharmacy* | | 1927 |
| **Maclaren, J. W. (Ed.)** | *Modern Trends in Diagnostic Radiology* | | 1948 |
| **Mettler, C. C.** | *History of Medicine* | | 1947 |
| **O'Connor, W. A.** | *Psychiatry—a short Treatise* | | 1948 |
| | *Preventive Aspects of Medicine* | (2) | 1934 |
| **Proetz, A. W.** | *The Displacement Method of Sinus Diagnosis and Treatment* | (4) | 1931 |
| **Roberts, F.** | *Medical Education* | | 1948 |
| **Rolleston, H. D.** | *Cambridge Medical School* | | 1932 |
| **Scherf, D. and Boyd, L. J.** | *Cardiovascular Diseases* | 2nd Ed. (5) | 1948 |
| **Schwartz, L. and others** | *Occupational Diseases of the Skin* | 2nd Ed. | 1947 |
| **Simpson, K.** | *Forensic Medicine* | (5) | 1947 |
| | *Sporotrichosis Infection on Mines of the Witwatersrand* | (8) | 1947 |
| **Stearns, H. O.** | *Elementary Medical Physics* | | 1947 |
| **Stone, J. E.** | *Secretarial Practice and Office Administration for Hospitals* | | 1947 |
| **Taylor, A. S.** | *Principles and Practice of Medical Jurisprudence* Ed. S. Smith, Vol. 1 | 10th Ed. | 1948 |
| **Ten Teachers** | *Midwifery* | 8th Ed. | 1948 |
| **Thoms, H.** | *Classical Contributions to Obstetrics and Gynecology* | | 1935 |
| **Tow, A.** | *Diseases of the Newborn* | | 1937 |
| **Weber, F. P.** | *Aspects of Death in Art and Epigram* | 2nd Ed. | 1914 |
| **Winton, F. R. and Bayliss, L. E.** | *Human Physiology* | 3rd Ed. | 1948 |

TRANSACTIONS, REPORTS, JOURNALS, ETC.

| | | |
|---|---|---|
| Dentists' Register 1948 | (1) | 1948 |
| List of Fellows and Members of the Royal College of Physicians 1947 | (6) | 1948 |
| Medical Directory, 1948. 2 Vols. | | 1948 |
| Medical Register, 1948 | (1) | 1948 |
| Proceedings of the University of Otago Medical School. Vol. 25 | (10) | 1948 |
| Report of the Henry Phipps Institute. Vol. 31, 1944-46 | (3) | 1947 |
| Yearbook of Endocrinology, Metabolism and Nutrition, 1947 | | 1947 |
| Yearbook of Neurology, Psychiatry and Neuro-Surgery, 1947 | | 1947 |
| Yearbook of Orthopedics and Traumatic Surgery, 1947 | | 1947 |
| Yearbook of Pathology and Clinical Pathology, 1947 | | 1947 |
| Yearbook of Physical Medicine, 1947 | | 1947 |

### Publications Received

FROM MESSRS. JOHN WRIGHT & SONS LTD.

*Physical Signs in Clinical Surgery.* By HAMILTON BAILEY, F.R.C.S., F.A.C.S. 11th Edition. Part 1. Price 8s. 6d. 1948.

*British Journal of Surgery.* Vol. 35, No. 140. April, 1948. Price 12s. 6d. Annual subscription 42s.

*An Index of Treatment.* By various writers. Edited by SIR ROBERT HUTCHISON, Bt., M.D., LL.D., F.R.C.P. assisted by REGINALD HILTON, M.A., M.D., F.R.C.P. 13th Edition. Price 84s. 1948.

*Emergency Surgery.* By HAMILTON BAILEY, F.R.C.S., F.A.C.S. 6th Edition. Part 2. Price 21s. 1948.

FROM GEOFFREY CUMBERLEGE, OXFORD UNIVERSITY PRESS :

*A Practical Manual of Diseases of the Chest.* By MAURICE DAVIDSON, M.A., M.D., F.R.C.P. 3rd Edition. Price 50s. 1948.

*Changing Disciplines.* By JOHN A. RYLE, M.D. Price 12s. 6d. 1948.

*Management in Obstetrics.* By ANDREW M. CLAYE, M.D., F.R.C.S., F.R.C.O.G. Price 12s. 6d. 1948.

FROM HODDER & STOUGHTON LTD. :

*Care of Tuberculosis in the Home.* By JAMES MAXWELL, M.D., F.R.C.P. 2nd Edition. Price 7s. 6d. 1948.

### Local Medical Notes

THE thirty-sixth Long Fox Memorial Lecture will be delivered in the University of Bristol on Tuesday, October 19th, 1948, by Professor R. Milnes-Walker, F.R.C.S., Professor of Surgery in the University of Bristol. Subject " Some Aspects of Hospital Economy ".

**University of Cambridge.**—The honorary degree of D.L. is to be conferred on Thomas Benjamin Davie, M.D., F.R.C.P., Principal and Vice-Chancellor of the University of Cape Town.

**Edward Fawcett Memorial Prize.**—The Edward Fawcett Memorial Prize for 1948 has been awarded to Mr. P. I. Draper.

**Thomas Francis Edgeworth and Francis Henry Edgeworth Prize.**—The Thomas Francis Edgeworth and Francis Henry Edgeworth Prize for the current session has been awarded to Miss June K. Lloyd.

THE FACULTY.

It is a tradition that the aristocrats destined for next day's guillotine dined festally in full dress and powder. In somewhat the same spirit the ancient Faculty of the Bristol Royal Hospital met for the last time on Wednesday, June 30th, 1948, at a dinner, as magnificent as present conditions allow, in the old Board Room of the Infirmary.

*Surgit amari aliquid!* There are no records of the first meeting but it was apparently held in 1738 : at least, from the earliest days, the Faculty met regularly for over 200 years, " to take order for the good conduct of the House ". The House—the very name is becoming obsolete, although it was regularly used by all members of the Royal Infirmary Faculty at least until 1920. We do not know yet who will constitute the new body nor what its name will be. But whatever its composition, work, hopes and achievements, it is impossible without regret to record the passing of the old Faculty.

**Medical Records Officers.**—The Association of Medical Records Officers held a week-end school at Bristol Royal Infirmary on May 29th–30th under the presidency of the Infirmary's House Governor, Mr. S. C. Merivale.

EXAMINATION RESULTS.

**University of Bristol.**—Students of the University have recently passed the following examinations :—

M.D.—*Dissertations Approved :* J. N. P. Davies (with Distinction), Cecil Mary Drillien.

M.D.—G. H. Wattley.

M.D.S.—*Dissertation Approved :* J. W. E. Snawdon.

M.B., Ch.B.—*First Examination :* D. L. Archer, G. A. J. Ayliffe, Prudence M. Beckingham, J. P. Bishop, Christine F. Boulter, T. A. V. Brooks, K. G. Collins, A. J. Coppen, M. L. Cox, R. Crossley, P. W. E. Downes, J. H. L. Griffin, Ruth L. Hickson, K. W. Moulding, A. E. Nesling, P. O. Nicholas, M. J. Page, E. C. Skeens, W. E. Smith, W. B. Spry, D. G. Trott, J. W. Ware, Angela C. Willis. *In Chemistry (completing the Examination) :* M. B. Gee. *In Physics (completing the Examination) :* Ruth J. Grose.

M.B., Ch.B.—*Second Examination :* Audrey M. Blake, C. T. Brown, J. P. Dymoke, Ann M. Haines, Audrey V. Hooper, R. J. Hunt, R. A. Iles, D. J. A. Jarvis, M. H. B. Joyce, June P. Lawson, Joan I. Light, Denise G. MacLeod, M. E. Parry, N. C. Tricks, Denorah Vardy, A. G. Walker, G. H. Wood.

M.B., Ch.B.—*Final Examination (Section I) :* Joan H. Barnes (with Distinction in Pathology and Bacteriology), B. B. Beeson, D. J. Brain,

Joan K. Cheeseman, Jennifer M. Comely, Diana F. J. Duncan, F. V. Eastman, A. R. Ebanks, P. C. Farrant, Margaret J. Francis, Elizabeth M. Fraser, D. M. Gambier, Patricia M. Gilbert, J. C. Gregg, Pamela Hawkins, Ruth E. Hazell, Pamela M. Hellings, G. Lucas, Ellen M. Marron, J. P. Martin, Elizabeth M. Morgan, June Morgan, P. A. Normandale, Lucy Rosenhaugh, H. Schnieden (with Distinction in Pharmacy, Pharmacology, etc.), J. D. Sheward (with Distinction in Pharmacy, Pharmacology, etc.), June M. Smith, Medora Storkey, M. P. Walker, G. Walters, Olwen J. West, Catherine C. White.

M.B., CH.B.—*Final Examination :* Barbara Brosnan, Suzanne K. R. Clarke, R. S. G. Davies, C. C. Downie, M. J. Dunn, Molly I. Gover, Pamela I. Hannaford, A. H. Levy (with Distinction in Forensic Medicine), Jenny Pym, F. A. A. Ruggeri, B. F. Vaughan, G. Winternitz, R. H. Wood. *In Group II* (*completing the Examination*): Elizabeth H. Chard. *In Group II only :* G. D. Teague, A. S. Wallace. *In Group I only :* Pamela L. C. Watson.

B.D.S.—*First Examination :* V. H. S. A'Court, C. E. Amos, A. H. P. Davies, H. W. A. Orton, M. D. Prosser, P. A. Watling.

B.D.S.—*Second Examination :* J. P. Fletcher, Joan M. Flint, R. L. Griffiths, L. K. James, S. W. James, J. G. J. Keene, A. N. Livingstone, R. T. Platt, Michalina B. Pyzik, A. Sander, R. K. M. Sanders, C. H. Thomas, J. K. Vowles, G. C. B. Winter.

B.D.S.—*Third Examination :* M. Bernstein, A. H. Chivers, Ruth A. Yearn.

B.D.S.—*Final Examination. In Section II* (*Dental Surgery*) (*completing the Examination*) *:* A. J. P. Cousins. *In Section I only :* L. M. Lloyd.

L.D.S.—*First Examination :* A. E. Lenko.

L.D.S.—*Second Examination :* M. F. Flint, A. H. Kingham, D. A. Pearce.

L.D.S.—*Third Examination :* E. K. Joseph, J. J. Tittle. *In Dental Anatomy* (*completing the Examination*) *:* G. D. Everard, H. T. Jones. *In Dental Mechanics* (*completing the Examination*) *:* Ruth Hellier. *In Dental Anatomy only :* R. H. Cross.

L.D.S.—*Final Examination. In Section II* (*Dental Surgery*) (*completing the Examination*) *:* M. J. Bartlett, Margaret J. Webb. *In Section I only :* J. P. B. Pengelly, H. Sheffield.

D.P.H.—*Part II :* R. G. Brennan, J. B. M. Davies, D. M. M. Jones, E. W. Moore, W. Nicol.

D.P.M.—*Part I :* M. Cocheme. *In Anatomy and Physiology only :* Muriel J. Davies. *In Psychology only :* K. W. Aron, H. A. Bowes.

D.P.M.—*Part II :* W. L. Jones, R. Maggs, J. R. Stuart.

D.M.R.D.—*Part I :* J. H. E. Bergin, W. R. Cole.

D.M.R.T.—*Part I :* K. Lal, H. H. Robinson.

D.M.R.T.—*Part II :* A. A. G. Flemming, F. A. Hanna.

M.R.C.S., L.R.C.P.—*Midwifery :* G. A Hanks, Una P. Jones, A. J. Lee, B. F. Vaughan. *Surgery :* Suzanne K. R. Clarke.

